# Induced premature ovarian insufficiency by using D galactose and its effects on reproductive profiles in small laboratory animals: a systematic review

**DOI:** 10.1186/s13048-019-0565-6

**Published:** 2019-10-16

**Authors:** Marzieh Rostami Dovom, Mahsa Noroozzadeh, Nariman Mosaffa, Azita Zadeh–Vakili, Abbas Piryaei, Fahimeh Ramezani Tehrani

**Affiliations:** 1 Reproductive Endocrinology Research Center, Research Institute for Endocrine Sciences, Shahid Beheshti University of Medical Sciences, N.24 Shahid Arabi st. Yaman Ave. Velenjak, Tehran, IR Iran; 2 Department of Immunology, School of Medicine, Shahid Beheshti University of Medical Sciences, Tehran, Iran; 3 Cellular and Molecular Endocrine Research Center, Research Institute for Endocrine Sciences, Shahid Beheshti University of Medical Sciences, Tehran, Iran; 4 Urogenital Stem Cell Research Center, Shahid Beheshti University of Medical Sciences, Tehran, Iran; 5 Department of Biology and Anatomical Sciences, School of Medicine, Shahid Beheshti University of Medical Sciences, Tehran, Iran

**Keywords:** Galactose, Premature ovarian insufficiency, Animal models, Systematic review

## Abstract

**Background:**

Development of a hyper-gonadotropic hypoestrogenism condition in women < 40 years, defined as premature ovarian insufficiency (POI), is the most common long-term complication in female survivors of galactosemia. In this systematic review, summarize the galactose (GAL) induced POI in rat and mice models.

**Methods:**

For this systematic review, we conducted a search of case control studies published from 1990 until August 2018 in PubMed/Medline, and Web of science, using the descriptors in the title/abstract field. A ‘pearl growing’ strategy was employed whereby, after obtaining the full text articles, reference lists of all included studies (*n* = 14) were reviewed for additional publications that could be used.

**Results:**

We selected and categorized 14 studies according to the time of exposure to GAL into two groups of prenatal (*n* = 4) and postnatal (*n* = 10). Findings of these studies showed that the different stages of follicular development are targeted differently by galactose exposure during the prenatal and postnatal periods: The small follicles (primordial and primary follicles) are targeted by galactose toxicity during prenatal exposure and the pre-antral and antral follicles are targeted by galactose toxicity during postnatal exposure.

**Conclusions:**

This systematic review shows that galactose has an ovotoxicity effect that can be used to induce appropriate POI animal models only if sufficient doses, proper onset time, and duration of prenatal exposure are taken into account. An optimized model of POI induction should manifest all the required ovarian morphological, hormonal, and estrus cycle changes.

## Introduction

Development of hyper-gonadotropic hypoestrogenism symptoms in women < 40 years of age is defined as premature ovarian failure (POF), a condition more recently referred to as “premature ovarian insufficiency” (POI) [1]. About 1% of general populations suffer from POI [2]. Concurrent estrogen secretory interruption from inner theca cells and granulosa cells in ovarian follicles predisposes these women to some morbidities such as ischemic heart disease, coronary heart disease, osteoporosis, cognitive impairment, and mood disorders [3–6]—adverse effects which occur at earlier ages in POI women. In 90% of cases the etiology of POI is unknown [7]; the well-documented factors genetic include abnormalities (Turner syndrome, fragile X syndrome), immunological disorders (Cushing syndrome, autoimmune thyroiditis), and enzymatic defects (galactosemia) [8]. POI can be the result of iatrogenic factors including radiotherapy, chemotherapy, and pelvic surgery [9, 10]. Cigarette smoking, chemicals, pesticides and viruses may also have detrimental effects on ovarian tissue, resulting in POI [11, 12].

POI can also occur due to follicular depletion or dysfunction [13, 14]. In the follicle depletion type of POI, women experience a progressive reduction in ovarian follicle pools that ultimately leads to complete ovarian follicular depletion. In the follicle dysfunction type of POI, oophoritis may occur; in some cases; however, despite the presence of very few primordial follicles—the main difference between POI and resistant ovary syndrome—the remaining follicles do not respond to gonadotropins, a condition described as resistant ovary syndrome (ROS) that may have a pathogenesis different from that of POI [15].

Despite the huge impact of POI on public health and quality of life of the women affected, its pathophysiology is uncertain, because of which POI cannot be prevented or cured yet. Animal models give us the opportunity to comprehensively explore the hypothesized pathogenesis. Although several efforts have been made to introduce an optimum model for POI, these models have several limitations. In some models POI was induced through oophorectomy, a model which cannot mimic spontaneous POI because the transitional period of ovarian depletion has been neglected.

Galactosemia—a rare genetic metabolic disorder that affects an individual’s ability to metabolize sugar galactose—is associated to POI. Lactose, the main sugar of milk and dairy products, is composed of two monosaccharides: galactose and glucose [16]. For galactose metabolism, three enzymes are involved in the Leloir pathway: galactokinase (GALK), galactose 1-phosphate uridylyltransferase (GALT), and UDP-galactose 4 epimerase (GALE) [17]; the last product of this pathway is UDP-glucose, which is used in glycation [18]. Another pathway in galactose metabolism is mediated by aldose reductase; the final product is galactitol, which is well documented to be involved in cataract formation [19]. POI is the most common long-term complication in survivors of galactosemia [20]. Accumulated galactose (GAL) in the absence of GALT in galactosemic women has an ovotoxic effect and finally leads to accelerated depletion in ovarian follicle reserve and the occurrence of POI [20]. Ovotoxicity, the hallmark of galactose, has led to the use of this monosaccharide to induce animal POI modeling interventions; however inducing an animal model using an ovotoxic agent with no serious damage to other organs is a challenging task.

So far several animal POI models using GAl have been introduced, each with some advantages and limitations, but none provide a comprehensive picture of POI. In this systematic review, we summarize these GAL-induced POF rat models.

## Materials and methods

**Fig. 1:**
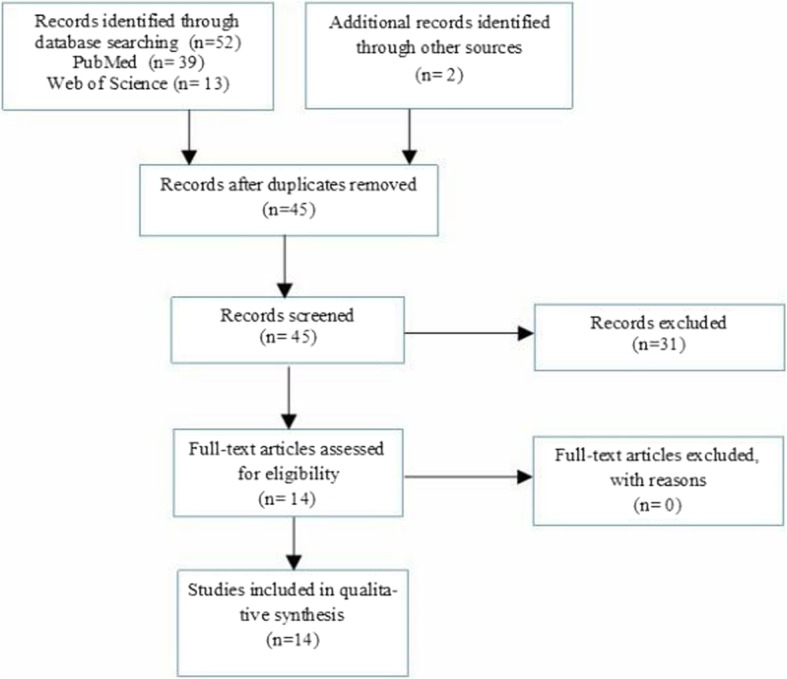
Flowchart of the search strategy for study selection

In this systematic review, we searched PubMed (1990–2018, and Web of Science (1990–2018) for relevant manuscripts, using the following keywords: “premature ovarian failure OR premature ovarian insufficiency OR primary ovarian insufficiency OR hyper gonadotropic ovarian failure OR gonadotropin resistant ovary syndrome OR premature menopause AND galactose OR D galactose AND animal OR animals AND model OR models” in the title/abstract field. Search limitations were other animals and publication languages other than English. Search strategies were almost similar for all databases, since they were conducted based on the ‘all fields’ in PubMed and ‘titles, abstracts and keywords’ in other databases. A ‘pearl growing’ strategy was employed whereby, after obtaining the full text articles, the reference lists of all studies included were reviewed for additional publications that could be used in this review. We excluded articles not exclusively related to the main objective of the present study, including data abstracts, review articles, editorials, letters, non-English manuscripts, and articles of rat models of POI induced by any other factors except GAL. Figure 1 illustrates the flowchart of the search strategy and study selection. Two reviewers (M.R.D and M.N.), in close consultation with the senior reviewer (F.R.T.), extracted data from full text articles; they double-checked all data extracted to minimize errors. For each study, the following information was extracted: number and appearance of ovarian follicles (primordial, primary, preantral, antral, atretic follicles, corpus luteum), hormonal findings, including ovarian follicles stimulating hormones (FSH), estrogen (E2), inhibin B, and anti-mullerian hormone (AMH); body weight, appearance, weight and size of ovaries, time of puberty (vaginal opening), and changes in estrus cycles.

### Quality assessment

We appraised all studies included for this systematic review for the quality of their methodological and result presentation using the gold standard publications checklist (GSPC) introduced by Hooijmans et al. [21]. Two reviewers (M.R.D. and M.N.), blinded to study authors, institution, and journal name, assessed the quality of the studies separately. Disagreements were resolved and settled by the senior reviewer (F.R.T.).

## Results

We selected and categorized 14 studies according to time of exposure to GAL into two groups: prenatal (*n* = 4) and postnatal (*n* = 10). Ten of the selected studies had developed their POI animal models by feeding them different percentages of GAL (20–50%); four studies had used injections of GAL (Table 1).

**Table 1:** Prenatally galactose-induced animal models for POI

Author, year (Refs.)	Strain & treatment	Gestational day of treatment group(s) & (age of sampling)	Results				
			Ovarian morphological histology	Hormonal profile	Estrus cycle, vaginal opening	Body weight	Metabolic disturbances
Chen et al., 1981 [22]	SD, Feeding by 50% galactose,	Grp 1 or prenatal Grp: 3rd to birth (at birth) Grp 2 or pre & postnatal Grp: 3rd to weaning (after weaning) Grp 3 or postnatal Grp: birth to weaning (after weaning) Grp 4 or post weaning Grp: 1–2 month (at 2 months) S-Grp 1:1st-9th ^(9th)^ S-Grp 2: 15th -20th S-Grp 3: entire gestational period S-Grp 4: 1st to weaning (Adv): pre and postnatal study	Grp1: there were no survivor Grp2:SF↓ (about 80%) Grp3:NSD in SF Grp4: MF & LF↓ S-Grp1:SF↓, MF NSD, LF NSD S-Grp2:SF↓, MF NSD, LF NSD S-grp3: SF↓, MF S-Grp 4: SF↓, MF NSD, LF NSD (about 50%)	NR (Lim)	S-Grp 1: DVO (+) S-Grp 2: DVO(−) S-Grp 3: NR S-Grp 4: NR	Grp 1:↓ Grp2: ↓↓ Grp3:↓↓ Grp4:less dramatic BW changes (+)	Grp1:GAL (+) Grp1:Gal-1-p (+) Grp3: GAL less than Grp 1 Grp3: Gal-1-p less than Grp 1 Control group: GAL & Gal-1-p undetectable
Bandyopadhyay et al., 2003 [23]	SD, Feeding by 35% galactose	Grp1: feeding GAL from 3rd through parturition (12–15 days of gestation from one uterine horn) Grp 2: feeding GAL from 3rd through parturition (PND 1 or 2)	Grp1: PGC↓	NR (Lim)	NR	NR	Grp 2: Liver epimerase activity ↓
Bandyopadhyay et al., 2003 [14]	SD, Grp1:, Feeding by 35% galactose Grp2: Feeding by 35% galactose and followed ovarian stimulating by PMSG-HCG between PND25/30, Grp3: Feeding by 35% galactose and followed desensitized by GnRH and induction after 10 days by PMSG-HCG	Feeding GAL from 3rd day of conception through weaning (PND21, 25/30, 42/43, 70/71)	AF absence CL absence SF↓ ATF↑	E2, PND 21↓ E2, PND 42/43↓ FSH, PND 21↑ FSH,PND 42/43↑	Delay VO(+)	Low BW, PND21: (+) GR, PND30/40: ↓	GAL: (PND21)↑ Gal-1-p: (PND21)↑ GAL: (PND70/71)↑ Gal-1-p: (PND70/71) ↑
Banerjee et al. 2012 [24]	SD, Grp 1: Feeding by 35% galactose, (PND 35) Grp 2: Feeding by 35% galactose, received subcutaneous (s.c.) injection of DES (2 mg/rat/day) for 4 days, or PMSG (25 mIU/rat/day) for 2 days. (PND 39–40)	Feeding from 3rd day of gestation and continuing through weaning of the litters.	Grp 1:ATF↑, bio activity GC: NC	Grp 1: FSH bioactivity↓	NR (Lim)	NR	Grp 1: GalTase ↓

*PND* Postnatal day, *Grp* Group, *S-Grp* Sub group, *(Adv)* Advantage, *(Lim)* Limitation, *FSH* Follicle stimulating hormone, *LH* Luteinizing hormone, *E2* Estradiol, *s.c*. Sub cutaneous, *PGC* Primordial germ cells, *PriF* Primary follicle, *PreAF* Preantral follicle, *AF* Antral follicle, *CL* Corpus luteum, *ATF* Atretic follicle, *GC* Granulosa cells, *NC* No change (no significant difference with controls), (−) = no observed, (+) = observed, ↑ = significant increase, ↓ = significant decrease, *NSD* No significant differences, *NSI* No significant increase, *SD* Sprague Dawley, *wks* weeks, *NR* Not reported, *BW* Body weight, *GR* Growth rate, *GAL* Galactose, *Gal-1-p* Galactose 1-phosphat, *Gat* Galactitol, *AlRd* Aldose reductase, *EC* Estrus cycle, *DVO* Vaginal opening, *SF* Small follicles (smallest oocyte surrounded by no more than a single layer of follicular cells), *MF* Medium follicles(growing oocytes surrounded by more than one layer of follicular cells and had no antrum), *LF* Large follicles (more mature antral follicles), *LEA* (Liver epimerase activity), GalTase = galactosyltransferase

### Prenatal galactose exposure models

Our search strategy finally retrieved four studies using prenatal-induced POI models following galactose exposure [14, 22–24]. Results, in terms of ovarian morphological changes, gonadotropins and sexual hormones, puberty manifestations, and changes in estrus cycles are summarized in Table 1. All of these models used feeding at different doses of GAL (35 to 50%).

#### Ovarian morphological changes

Results of the studies show that exposure to galactose during the embryonic period reduces the number of primordial and primary follicles and alters the development of the ovaries by two pathways: increase in the number of apoptotic cells and decrease immigration of primordial germ cells (PGC) [14, 22–24].

It has been shown that feeding galactose (35%) from the third day of pregnancy till the end of the lactation period increases the number of apoptotic follicles in the ovaries of pups and that the toxic effect of galactose disrupts the migration of germ cells from the yolk sac to the gonads, and the intervention group basically faces poor ovarian reserves [23]. Compared to controls, there was no significant difference in the number of preantral and antral follicles in GAL prenatal exposure [22]. The greatest reduction in the number of small follicles occurred when the galactose intervention was implemented before the fifteenth day of pregnancy, before the end of the meiosis period, because intervention after the end of meiosis does not affect the number of small follicles [22]. Therefore, the premeiotic stages (< 16 ½ days of gestational period) of oogenesis are the most critical period for changes in oocyte numbers. The decrease in oocyte numbers is the result of attenuation in the migration, proliferation, and differentiation of primordial germ cells [22, 23]. Since prenatal exposure to galactose before the 15th day of the embryonic period reduces the number of primordial germ cells’ migration to the future sites of gonads, female pups are born with reduced ovarian follicle reserve [23]. Ovulation is affected by galactose exposure during the prenatal and weaning periods; the low number of *corpus luteum* and Graafian follicles are a result of a decrease in primordial follicles and an increase in the number of atretic follicles [14].

#### Biochemical and metabolic changes

Two studies reported an increase in the levels of galactose and galactose 1-phosphate in female pups prenatally exposed to galactose [14, 22]; although this increase was observed in those exposed in the weaning and gestational periods, it was less prominent in the embryonic period [22]. Liver epimerase and GalTase activity were also reduced in the galactose exposure group [23, 24].

#### Hormonal changes

Hormonal changes were investigated in only two prenatal GAL exposure studies [14, 24] that reported that this exposure was associated with an increase in serum FSH and a decrease in estrogen levels; moreover, FSH bioactivity in the galactose feeding group was decreased despite no changes in the bioactivity of granulosa cells [25].

#### Growth rate, vaginal opening, & estrus cycle changes

Regardless of the time of exposure [14, 24], prenatal exposure to GAL during both the embryonic and weaning periods disrupted the growth rate and were more worsened than during the embryonic period [22]. Delays in vaginal opening were reported in a group of infants exposed to galactose from the 3rd to the 9th days of gestational age, a delay however not observed in the group exposed to galactose from the 15th day of pregnancy [22]; another study reported delays in opening of the vagina exposed to galactose from the third day to weaning [14].

Changes in estrous cycles have not been investigated in any study of galactose exposure that had occurred during the prenatal period.

### Postnatal galactose exposure models

We retrieved 10 related studies on postnatal interventions using galactose for POI induction and their findings are summarized in Table 2. Data on postnatal GAL are inconclusive as different mice, different doses, and methods of use of galactose (feeding/injection) were used to induce the POI models. Since the breed and the dose used in studies were not the same, the results of each study have been reported separately.

**Table 2:** Postnatally galactose-induced animal models for POI

Author, year (Refs.)	Strain & treatment	Age at treatment in group (s) & (age of sampling)	Results					
			Ovarian morphological histology	Appearance changes	Hormonal profile	Estrus cycle, vaginal opening	Body weight	Metabolic disturbances
Swartz et al., 1988 [26]	White mice, feeding by 50% galactose, superovulation by HMG, and HCG	6 weeks, Grp1:feeding by galactose for two weeks (8 weeks) Grp 2: feeding by galactose for four weeks (10 weeks) Grp 3: feeding by galactose for six weeks (12 weeks) Grp 4: Feeding for 2,4, and 6 weeks, then superovulation (the day after treatment) Grp 5: Feeding for 7 weeks, then regular standard feeding for 1 week, then super-ovulation (the day after treatment) (Adv)	Grp1,2,3: CL↓ Grp 4: CL↓, ovulatory response ↓ Grp 5: ovulatory response (NC)	NR	NR (Lim)	NR (Lim)	Grp 1,2,3,5↓ Grp 4: NR	NR
Yan z et al. 2018 [27]	C57BL/6 female mice, aged, subcutaneously injected D galactose 200 mg/kg/day for 42 days,	7–8 weeks (after 42 days intervention)	PMF ↓ AGC ↑	NR	FSH↑ LH↑ E2↓ P↓ AMH mRNA expression ↓ (ADV)	NR (Lim)	NR	NR
Zhang et al. 2016 [28]	KM mice, (age?) feeding 35% galactose, for 70 days	PND?,(Lim) (70 days after feeding)	OO (NEC) GC (APOP)↑ CL↓ No mature oocyte in antral follicles	No abnormality in uterus No obvious pathological change in ovary	FSH↓ LH (NSD) E2 (NSD)	NR (Lim)	GR↓ BW↓	GAL↑ Alb↑ TP↑
Zhang et al. 2017 [29]	ICR F1 hybrids, 12–14 days, preantral follicle culture, 5 mg/ml D-galactose. **Culture and in vitro**	Only preantral follicles with two layers of granulosa cells (100–130 μm) with intact basal membrane and some theca cells	Inhibit follicle development it does not exhibit obvious follicular toxicity at day 2 of culture, the influence of D-galactose on oocyte maturation is relatively weak	NR	NR (Lim)	NR (Lim)	NR	NR
Meyer et al. 1992 [30]	SD rat, feeding by 40% galactose	Pre pubertal,PND 21–24, (immediately after weaning)	ovulation ↓ granulosa cells↓	NR	NR (Lim)	NR	Body weight↓ Ovary weight↓	Ovarian Galactitol concentration↑
Ahangarpour et al. 2016 [31]	NMRI mice, injected subcutaneously with D-galactose 500 mg/kg/d for 45 days) and concomitantly administered normal saline by gavage twice daily for the last 7 days	3 months (after 45 days treatment)	No Graafian follicles No *zona pellucida*	Atrophy of the endometrium	LH↑ FSH↑ E2↓ progesterone↓	Disrupt the estrous cycle	Body weight↑ Ovary and uterus -weight/body weight↓	NR
Wang et al. 2018 [32]	Kunming white mice, Daily ip. Injection of 200 mg/kg D-galactose (dissolved in saline, 0.2 ml/mouse/day) for 50 days, and between the 21st to 50th day of D-galactose injections, the group also received 0.5% CMC-Na solution (0.3 ml/mouse/day) by gavage.	Six to eight week-old (the end of 50 days treatment)	No mature follicles More atretic follicles Reduced follicle numbers Smaller follicles Diameter of ovarian follicles at all stages ↓ Proportion of secondary follicles ↑ The number of interna theca cells in tertiary follicles ↓ The number of externa theca cells in tertiary follicles ↑	NR	FSH↑ LH↑ AMH↓ E2↓	NR (Lim)	NR (Lim)	NR
Liu et al. 2005 [33]	Long-Evans rats, High lactose diet (HLD),41.9 g Lactose Ovaries morphology at 2 months of age for baseline, seven months later for intervention outcomes	25-days-old, (7 month later)	No significant differences in the number of primordial, growing, and antral follicles		Progesterone ↓ Estradiol (NC)	Irregular EC from 5 months of age	Body weight ↓ Increased fresh ovary weight (+)	NR
Liu et al. 2006 [34]	Long-Evans rats, 20% galactose diet,	21-day-old,(PND 40)	Growing and antral follicles ↓ Primordial NC Atretic NC Non growing follicles ↑	NR (Lim)	no significant changes in the serum concentrations of estradiol and progesterone	The study has done before the first spontaneous ovulation (ADV)	Body weight (NC) Ovaries w (NC) Uterus weight ↓	NR
Lai et al. 2003 [35]	SD, Feeding by 50% galactose for 4 weeks, followed by PMSG (50 IU/100 g) and HCG (50 IU/100 g)	3–4 weeks, Grp i: 4 weeks GAL diet, PMSG (48 h later) Grp ii: 4 weeks GAL diet, PMSG, 48 h later HCG (8 h later) Grp iii: 4 weeks GAL diet, PMSG, 48 h later HCG (13 h later) Grp iv: 4 weeks GAL diet, PMSG, 48 h later HCG (18 h later)	Ovulated OO↓ *corpora lutea* ↓ volume *corpora lutea*↑	NR (Lim)	NR (Lim)	NR (Lim)	BW↓ GR↓ Kidney W↑ Spleen w↑ Liver↑ ovarian and uterus (NC)	galactose-1-phosphate↑

*PND* Postnatal day, *Grp* Group, *S-Grp* Sub group, *(Adv)* Advantage, *(Lim)* Limitation, *FSH* Follicle stimulating hormone, *LH* Luteinizing hormone, *E2* Estradiol, *P* Progesterone, *s.c.* Sub cutaneous, *PGC* Primordial germ cells, *PMF* Primordial follicles, *PriF* Primary follicle, *PreAF* Preantral follicle, *AF* Antral follicle, *CL* Corpus luteum, *ATF* Atretic follicle, *GC* Granulosa cells, *AGC* Atretic granulosa cells, *NC* No change (no significant difference with controls), (−) = no observed, (+) = observed, ↑ = significant increase, ↓ = significant decrease, *NSD* No significant differences, *NSI* No significant increase, *SD* Sprague Dawley, *wks* Weeks, *NR* Not reported, *BW* Body weight, *GR* Growth rate, *GAL* Galactose, *Gal-1-p* Galactose 1-phosphat, *Gat* Galactitol, *AlRd* Aldose reductase, *Alb* Albumin, *TP* Total protein, *EC* Estrus cycle, *VO* Vaginal opening, *SF* Small follicles (smallest oocyte surrounded by no more than a single layer of follicular cells), *MF* Medium follicles(growing oocytes surrounded by more than one layer of follicular cells and had no antrum), *LF* Large follicles (more mature antral follicles), *OO* Oocyte, *NEC* Necrosis, *APOP* Apoptosis, *LEA* (Liver epimerase activity), *GalTase* Galactosyltransferase

#### Ovarian morphological changes

Feeding with galactose (35 to 50%) reduces the number of *corpora lutea* as an ovulation index [26, 28, 35]. Despite not mentioning the time of administration of GAL in one study [28], all mice aged 4 to 6 weeks that were exposed to galactose for 2 to 6 week, suffered reduced ovulation [26, 30, 35]. In a group of mice fed with galactose for 7 weeks, ovulation returned to normal status 1 week after receiving standard food; the findings indicated that galactose effects on ovulation had been temporarily delayed and that ovulation had returned to normal with galactose-free feeding.

The side effects of galactose activity on growing follicles included the following: reduction in the number of granulosa cells [30], with an increase in the number of atretic granulose cells [27, 28], absence of Graafian follicles without the presence of zona pellucida [31], or the presence of a greater number of small diameter follicles, and reduction of internal theca cells along with an increase of external theca cells [32]. In two studies the apoptosis numbers in granulosa cells increased following feeding of 35% galactose [28], a finding not observed with feeding of 20% galactose [34],; neither did the use of high-lactose food as a source of galactose have similar effects on ovarian follicles [33]. The effect of galactose on the growth of oocytes in cell culture was not found to be significantly different from the control group the effects were relatively poor [29].

#### Biochemical and metabolic changes

The effects of galactose feeding on different biochemical parameters including galactose, galactose 1-phosphate, galactitol, albumin, and total protein levels have been investigated in three studies [28, 30, 35], all of which reported that these parameters and ovarian galactitol content were significantly higher in the galactose intervention groups than in control groups.

#### Hormonal changes

The hormonal profiles of galactose exposure have been investigated in several studies [22, 27, 30–32], that reported this exposure to be associated with a significant increase in gonadotropins, FSH, and LH [27, 31, 32], and with a decrease in estradiol [27, 30, 32]. Exposure to 20% galactose did not change the serum estradiol and progesterone levels, indicating that the hormone changes were also dependent on galactose doses [34]. Surprisingly, the only study reporting a reduction in serum FSH levels was the Zhang et al. study [28] in which feeding animals 35% galactose not only reduced serum FSH levels but did not show any changes in serum estradiol and LH; no justification for this finding was reported in the study.

Changes in the level of anti-mullerian hormone (AMH) as an indicator of the ovarian follicular reserve was investigated in a single postnatal study [27], the results of which found that in comparison to the control group, mRNA expression of AMH in the study group was significantly reduced.

#### Growth rate, vaginal opening, & estrus cycle changes

Body weight loss and decline in growth rates in galactose-exposed animals have been reported in most studies [28, 30, 33, 35]; however, in one study [31], weight gain was reported in mice receiving 500 mg/kg/day for 45 days of galactose injection; a major difference in this study was the age of initiation of intervention in 3-month-old mice that received injectable galactose for 45 days.

Only two studies examined the estrus cycles of postnatal animals under galactose exposure; both studies reported changes in estrus cycles [31, 33], which were disrupted in mice aged ≥3 months.

## Discussion

This study summarizes the current evidence available on the biochemical, hormonal, morphological, and clinical manifestations of prenatal/postnatal exposure of rats and mice to galactose. It should be noted that all these studies were performed on animals that did not have any enzyme deficiency. Findings of these studies showed that the different stages of follicle development are targeted differently by galactose exposure during prenatal and postnatal periods; small follicles (primordial and primary follicles) are targeted by galactose toxicity during prenatal exposure; the pre-antral and antral follicles are battered during postnatal galactose exposure.

A review of oogenesis and folliculogenesis suggests that the evolution of the human reproductive system initiates from the fifth week of the gestational period with the migration of germ cells from the yolk sac to the future gonadal ridge and their transformation to oogonia. The increase in numbers of oogonia continues with mitotic divisions, and after reaching the right number (around the third month of pregnancy) they transformed to the primary oocytes. After completing the synapses and recombining the divisions, the divisions cease and do not go through the remaining stages of meiosis. The primary oocyte consists of a flat cell line derived from the ovarian stroma located around the nucleus. Human females receive a finite pool of follicles during fetal development. Early accelerated depletion of ovarian follicles may result in the reduction of follicular ovarian reserve due to decreased migration of germ cells to the gonads or accelerated apoptosis rates of follicles. Both hypothesis have been investigated in animal models during different prenatal or postnatal periods in experimental studies.

Galactose exposure applies its destructive effect on oocytes through several pathways; apparently, folliculogenesis from initiation to growth development is affected by galactose. Prenatal galactose exposure disrupts the migration of primordial follicles during the embryonic period [23]. In mice and rats, primordial germ cells arrive at the genital ridge and are divided by mitosis until around 13.5 days postcoitum [36]; exposure to galactose from the third day of pregnancy in rats can lead to a decrease in the number of migrating follicles to the gonadal area, resulting in poor ovarian reserves in female pups; bioactivity of FSH is also reduced by galactose toxicity [25], subsequently reducing the number of growing follicles and resulting in decreased production of estradiol by the granulosa cells, similar to that observed in POI women. Follicular growth disturbances that are characterized by (1) decrease in theca internal cells, (2) increase in the number of theca external cells, and (3) destruction of the *zona pellucida*, under the influence of galactose in the first months after birth, lead to a decrease in the number of follicles and an increase in the number of non-effective small follicles. This results in a lack of mature follicles and subsequently in estrogen production deficiency; it seems that primordial follicles during fetal development are targets of galactose toxicity; the attenuation in follicle development during this period leads to the absence of antral follicles and ovulation in the following stages [32].

The ovotoxicity effect of galactose on oocytes during postnatal interventions is most probably applied through down-regulation of the growth differentiation factor-9 (GDF-9) in the rat ovary [34]. Data provide strong evidence that follicle growth is suppressed by autocrine and paracrine products derived from oocytes and granulosa cells; the growth differentiation factor-9 (GDF-9), which is expressed in oocytes, plays a main role in the gonadotropin-independent phase in folliculogenesis [37]. High galactose feeding after weaning, but before onset of the first spontaneous estrus cycle, down-regulates the GDF-9 expression in follicles and inhibits follicular development [34].

Another hypothesis for early reduction of ovarian follicles is the increase in follicular apoptosis, which is a normal process for removing excess ovarian cells and is necessary for ovary development [38]. Granulosa cells are the major cell types undergoing apoptosis in ovarian follicles [38]. Induced apoptosis in experimental in vivo and in vitro studies successfully designed by deprivation of gonadotropins result in atretic changes in large antral follicles [39]. Estrogens play a very important role in the regulation of the death of ovarian cells by preventing apoptosis [40]. Estrogen and androgen production decrease in the atretic follicles of rats [41]. In both pre-natal and post-natal studies, the induction with 35% galactose (but not 20%) increases apoptosis in granulosa cells and decreases the rate of estrogen secretion [24, 34]. However, in one study, despite an increase in apoptosis induced by galactose, no decrease in estrogen levels were observed [28]. By increasing apoptotic follicles and reducing estrogen production, galactose toxicity plays a major role in the development of primary ovarian insufficiency due to early depletion of ovarian follicles.

In female rats and mice the correct time of puberty (vaginal opening, estrus cycle, and ovulation) is dependent on the normal development of the hypothalamic- pituitary-ovarian axis [42]. The vaginal opening in rats is affected by various variables, including environmental factors, hormones, and body weight to the extent that in mice with body weight < 60 g, the vaginal opening usually does not occur [43]. Early underfeeding by high galactose may change both fetal programming and organ development [44]. Losing body weight (galactose induced) both in prenatal and postnatal experiments has been reported, whereas vaginal opening was delayed only in those animals prenatally exposed to galactose between days 1–9 [22], indicating that weight loss may not be the only factor in the delay of vaginal opening and probably other factors, including endocrine disrupters (followed by galactose toxic effects on the ovary) are involved.

## Conclusion

Galactose has an ovotoxicity effect if administered in sufficient doses, at proper onset times and duration of prenatal exposure, and can be used to induce appropriate POI animal models. An optimized model of POI induction should manifest all the required ovarian morphological, hormonal, and estrus cycle changes.

## Data Availability

All relevant data are within the paper.

## Abbreviations

AMH: Anti mullerian hormone
E2: Estrogen
FSH: Follicles stimulating hormones
GAL: Galactose
GALE: UDP-galactose 4 epimerase
GALK: Galactokinase
GALT: Galactose 1-phosphate uridylyltransferase
GDF-9: Growth differentiation factor-9
GSPC: Gold standard publications checklist
PGC: Primordial germ cells
POF: Premature ovarian failure
POI: Premature ovarian insufficiency
ROS: Resistant ovary syndrome
